# Advancing the aging biology toolkit

**DOI:** 10.7554/eLife.42976

**Published:** 2018-11-28

**Authors:** Troy K Coody, Adam L Hughes

**Affiliations:** Department of BiochemistryUniversity of Utah School of MedicineSalt Lake CityUnited States

**Keywords:** ATAC-Seq, ageing, Miniature-chemostat Aging Device, yeast, longevity, gene expression, *S. cerevisiae*

## Abstract

A new device for isolating large quantities of old yeast cells expands the experimental boundaries of aging research.

**Related research article** Hendrickson DG, Soifer I, Wranik BJ, Kim G, Robles M, Gibney PA, McIsaac RS. 2018. A new experimental platform facilitates assessment of the transcriptional and chromatin landscapes of aging yeast. *eLife* **7**:e39911. doi: 10.7554/eLife.39911

Aging is a universal feature of life. It occurs at the level of both cells and organisms, and is the single greatest risk factor for disease. Researchers have been working to unlock the mysteries of aging for decades, and have identified several key molecular changes that drive age-associated traits, as well as genetic, pharmacological and metabolic changes that control lifespan. Because aging is a complex and lengthy process, most breakthroughs have come from studies on model organisms with short lifespans, including yeast, flies, worms and mice. Remarkably, these studies have shown that age-associated traits and genes regulating lifespan are highly conserved, raising the hope that therapeutic interventions that target aging are a real possibility in the near future (Bitto et al., 2015).

Of all these model systems, the budding yeast, *Saccharomyces cerevisiae*, is the simplest, and has been used to study aging since the 1950s. At that time, Robert Mortimer and John Johnston used microdissection, a technique that involves separating yeast daughter cells from their mothers after they divide, to demonstrate that yeast undergo a finite number of divisions before they die (Mortimer and Johnston, 1959). This type of aging is called replicative aging, and it is defined by the number of times an individual yeast cell asymmetrically divides to produce a daughter. Since then, researchers have used yeast to uncover a number of age-associated traits and genetic modifiers of lifespan (Wasko and Kaeberlein, 2014).

Despite the many successes of yeast-aging research, the field has always faced a significant challenge: old yeast cells are exceedingly rare in a growing population. Early on, this obstacle limited the experimental approaches researchers used, because they could not obtain enough old cells for analysis. Over the years, several laboratories have made key technical advances that have enabled the field to overcome this obstacle and harness a larger spectrum of techniques beyond microdissection to identify molecular mechanisms associated with aging (Figure 1).

**Figure 1.**
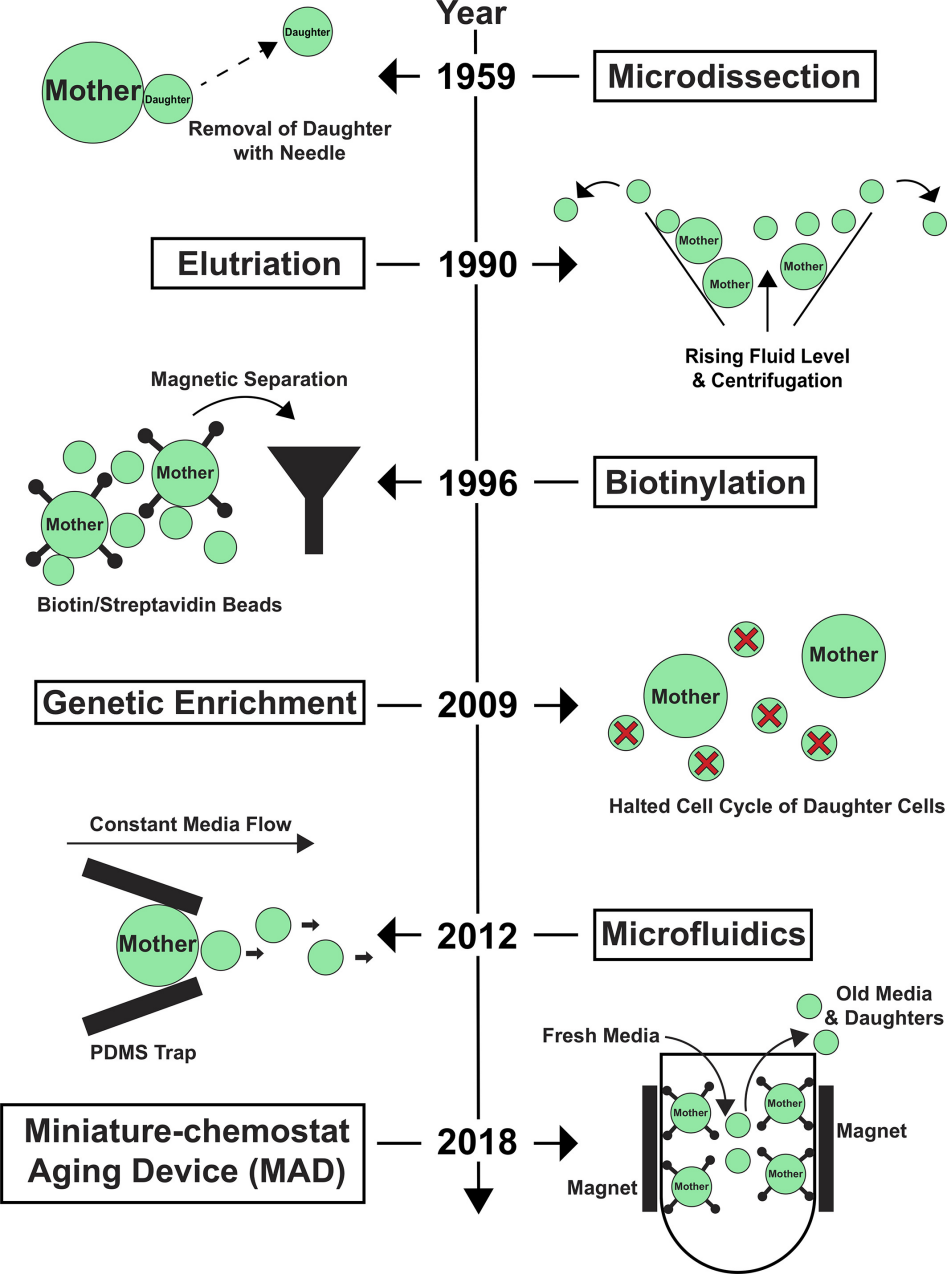
Key technological advances in yeast-aging research. The development of new tools to study replicative aging in yeast has been crucial to overcome the limitations imposed by the scarcity of old yeast cells in a growing cell population. This timeline depicts broadly adopted technologies that have enabled both single-cell and large-scale measurements using biochemical or genetic approaches to characterize the molecular mechanisms of aging; see main text for more details. Large circles represent mother yeast cells; small circles represent daughter yeast cells; small circles with a red cross represent daughter cells prevented from maturing.

These developments have included: i) microfluidic imaging devices that enable continuous imaging of individual yeast cells over their lifespan (Chen et al., 2017); ii) centrifugation-based approaches that separate populations of old mother cells from young daughters based on size (elutriation; Egilmez et al., 1990); iii) large-scale isolation of aged mother cells by attaching biotin to their cell wall prior to aging (a process known as biotinylation), and then using magnetic microbeads coated with the protein streptavidin to magnetically separate the biotinylated mother cells from their daughters (Smeal et al., 1996); iv) genetic enrichment of aged mother cells by stopping newborn daughter cells from growing (Lindstrom and Gottschling, 2009).

Combined, these techniques have pushed the yeast-aging field to new heights. However, each method has its limitations. For example, while microfluidic devices permit constant media exchange during aging, they are limited to single-cell analysis. On the other hand, genetic enrichment combined with biotin-based purification strategies allows researchers to isolate large numbers of aged cells for a range of analyses. However, this system requires genetically modified yeast strains and does not allow rapid and continuous media flow.

Now, in eLife, Scott McIsaac and colleagues at Calico Life Sciences – including David Hendrickson as first author – report that they have engineered a new aging platform, called the Miniature-chemostat Aging Device (MAD), which pushes the capabilities of the yeast-aging field one step further (Hendrickson et al., 2018). This new device helps to isolate large numbers of yeast cells across a range of ages and genetic backgrounds without the use of genetically modified systems, but with the benefit of continuously renewed media.

Hendrickson et al. achieved this by combining the Miniature-chemostat (Miller et al., 2013) with magnetic-based streptavidin enrichment of mother cells. The MAD approach worked as follows: cells were biotinylated and attached to streptavidin beads prior to aging. The bead-conjugated cells were then loaded into culture tubes fitted with neodymium ring magnets, which trapped the mother cells along the vessel walls, while allowing the daughter cells to be released. The device was connected to a peristaltic pump, which provided fresh media to the confined mother cells while washing away daughters. Mother cells could be released from the magnet at any point during the aging process, and collected for further analysis.

Hendrickson et al. put their new device to the test, performing several genetic and molecular techniques on purified yeast mother cells of various ages and genetic backgrounds. The results confirmed previous observations that aging in yeast is associated with an activation of the core environmental stress response (a set of genes that respond to stress) and the accumulation of ribosomal DNA transcripts (Sinclair and Guarente, 1997; Lesur and Campbell, 2004). They also demonstrated the tremendous potential of this new device to identify unknown age-associated traits by showing that origins of replication (sites were the replication of DNA is initiated) become less accessible with age, and that gene expression from sub-telomeric regions (regions near the end of the chromosomes) increases with age. Moreover, Hendrickson et al. challenged previous observations in the field that global nucleosome occupancy (the density of nucleosomes on DNA) declines with age (Hu et al., 2014).

Overall, yeast-aging research has come a long way since the pioneering studies of Mortimer and Johnston. While there are still significant hurdles to overcome, the development of MAD opens an exciting new era for yeast-aging research.
